# RE-EXAMINING PATIENT-CENTERED CARE THROUGH DESIGN PROCESS

**DOI:** 10.1093/geroni/igac059.612

**Published:** 2022-12-20

**Authors:** Kristine Mulhorn, Shushi Yoshinaga

**Affiliations:** Drexel University, Wallingford, Pennsylvania, United States; Drexel University, Philadelphia, Pennsylvania, United States

## Abstract

Service design is the adoption of design process to healthcare and other service sectors. This was a transdisciplinary research project in which investigators were faculty members from Graphic Design and Health Administration. In addition, two student research assistants were recruited from undergraduate Graphic Design and Health Science majors. The objective of service design is to involve consumers, designers, and businesspeople in an integrative process, which can be applied to post-acute rehabilitation hospital settings focusing specifically on the experience of those who are 65 and older. The aim of this pilot study was to explore “designing with people rather than just for them”, an approach to improve the patient experience. Our first step involved on-site interviews. The patient narratives raised challenges and positive aspects of their interactions with the facility. On our initial site visit, we interviewed five members of clinicians and administrative staff. During the two follow-up visits, our student research assistants interviewed seven patients. Based on our staff member interview findings, we developed a revised set of questions for patients. The questionnaire was divided into three sections related to appointments: pre-arrival, during the visit, and after their appointments. Interview results were summarized in a visual data format and collaborative recommendations were made during the final presentation such as interior layout, wayfinding, online portal and their functionalities. Our findings also confirmed that the interior signage created confusion, promoting frequent questions to staff. These results will engage stakeholders and contribute to a co-designing process that will ultimately improve the patient journey.

